# Vitamin D and early rheumatoid arthritis

**DOI:** 10.1186/s41927-020-00134-7

**Published:** 2020-07-27

**Authors:** Stephanie R. Harrison, Gurpreet Jutley, Danyang Li, Ilfita Sahbudin, Andrew Filer, Martin Hewison, Karim Raza

**Affiliations:** 1 Institute of Metabolism and Systems Research, University of Birmingham, Birmingham, B15 2TT UK; 2 Department of Rheumatology, Sandwell and West Birmingham NHS Trust, Birmingham, B18 7QH UK; 3 Institute of Inflammation and Ageing, Research into Inflammatory Arthritis Centre Versus Arthritis and MRC Versus Arthritis Centre for Musculoskeletal Ageing Research, University of Birmingham, Birmingham, B15 2TT UK; 4 Centre for Endocrinology, Diabetes and Metabolism, Birmingham Health Partners, Birmingham, B15 2TT UK

**Keywords:** Vitamin D, Inflammation, Rheumatoid arthritis, Psoriatic arthritis, Undifferentiated inflammatory arthritis

## Abstract

**Background:**

Previous studies have linked rheumatoid arthritis (RA) risk and disease activity with vitamin D-deficiency (low serum 25-hydroxyvitamin D (25OHD)), but a causal role for vitamin D in RA is still unclear, with conflicting results from many previous studies, partly due to heterogeneity in study design and patient populations. In this study we aimed to (1) analyse serum 25OHD in early inflammatory arthritis, (2) compare 25OHD with disease activity and fatigue in early RA and (3) determine whether low 25OHD is associated with progression to RA.

**Methods:**

An analysis of 790 patients recruited to the Birmingham Early Inflammatory Arthritis Cohort and followed longitudinally to determine clinical outcomes. The following were recorded at baseline: demographic data, duration of symptoms, duration of early morning stiffness (EMS), tender and swollen joint counts, Visual Analogue Scale (VAS) pain/fatigue/EMS, PHQ-9, HAQ and FACIT-Fatigue scores, DAS28-ESR, DAS28-CRP, CRP, ESR, anti-CCP antibody status, rheumatoid factor status, and serum 25OHD (ng/ml). Diagnosis was recorded at 0 and 12 months onwards as either RA, Undifferentiated Inflammatory Arthritis (UIA; synovitis not meeting other classification/diagnostic criteria), Clinically Suspect Arthralgia (CSA; arthralgia of an inflammatory type without synovitis), or Other.

**Results:**

Baseline demographic data were similar between all groups, with median symptom duration of 16.8–34.0 days. Baseline 25OHD was not significantly different between groups [median, interquartile range (IQR): RA 46.7, 30.0–73.3; UIA 51.4, 30.0–72.3; CSA 47.7, 30.3–73.0; Other 39.9, 28.6–62.2]. In RA (*n* = 335), there were no significant differences between 25OHD and measures of disease activity or fatigue. No association between 25OHD and progression from UIA or CSA to RA was observed.

**Conclusions:**

There was no clear association between serum 25OHD and baseline diagnosis, RA disease activity, or progression from UIA or CSA to RA. Future studies of other vitamin D metabolites may better define the complex role of vitamin D in RA.

## Background

Rheumatoid arthritis (RA) is a chronic inflammatory disease characterised by synovitis affecting the small/medium joints and extra-articular manifestations [1]. Early diagnosis and treatment maximise chances of inducing remission thereby preventing permanent disabling joint damage [2]. Currently there are few clinical or biochemical markers to predict who will respond to RA treatment, and approaches to predicting which patients with undifferentiated inflammatory arthritis (UIA) or clinically suspect arthralgia (CSA) will progress to RA are imperfect [3, 4]. However, recent studies have suggested that vitamin D status may be a potential contributor to inflammatory disorders such as RA [5]. In vitro, vitamin D metabolites modulate inflammation through changes in T helper and regulatory T cell function [5, 6]. In vivo, vitamin D metabolites including the main circulating metabolite 25-hydroxyvitamin D (25OHD), and the active form of vitamin D 1,25-dihydroxyvitamin D (1,25(OH)_2_D) have been linked to RA disease progression [7–10], and vitamin D supplementation may have beneficial effects for patients with established RA [11, 12]. What is less clear is the role of vitamin D status in the onset and early progression of RA. In 2015, Cooles et al. reported no relationship between 25OHD and CRP, ESR, symptom duration, tender joint count (TJC), swollen joint count (SJC), or patients’ global health visual analogue scale (GH-VAS) in a small cohort of 73 RA patients [13], and similarly Neilen et al. found no evidence of low vitamin D in blood donor samples from patients in the pre-RA phase [14]. Conversely, other studies have reported an association of serum 25OHD or 1,25(OH)_2_D status with some markers of disease activity in early RA [15, 16].

The aim of the current study was to use a large prospective cohort of early inflammatory arthritis patients to: (i) compare total serum 25OHD levels between early inflammatory arthritis patients with different baseline diagnoses; (ii) explore the relationship between 25OHD levels and measures of RA disease activity and of fatigue, and; (iii) determine whether low serum 25OHD is linked to progression from CSA or UIA to RA.

## Methods

We enrolled patients with inflammatory arthritis or CSA newly seen in rheumatology clinics into the Birmingham Early Inflammatory Arthritis cohort (BEACON) between 2013 and 2019 from rheumatology outpatient departments at Sandwell and West Birmingham NHS Trust and University Hospitals Birmingham NHS Trust. Demographic information including age, sex, BMI, smoking status and ethnicity were recorded. In addition, clinical data including duration of symptoms, early morning stiffness (EMS), Tender Joint Count (TJC) and Swollen Joint Count (SJC), DAS28-ESR, DAS28-CRP, visual analogue scale (VAS) pain, fatigue and EMS, PHQ9, HAQ and FACIT-Fatigue scores were collected, alongside CRP, ESR, anti-CCP antibody and RF levels, at baseline (0 months (M)). Classification criteria for RA [17] were applied at 0 M and at 12 M or beyond. In addition, we looked at two distinct groups of patients representing different time points in the transition to RA. Patients with synovitis not meeting criteria for RA or other rheumatological diseases (i.e. UIA) and inflammatory-type arthralgia without synovitis (CSA). Serum 25OHD concentrations were analysed using NHS laboratory assays and reported as ng/mL.

Box-and-whisker diagrams depicting the median and interquartile range (IQR) were used to describe non-parametric variables, and percentages for categorical variables. Significance between groups was determined by non-parametric testing with either Pearson’s correlation coefficient, Kruskal-Wallis test and Chi-squared tests as appropriate, using SPSS v25. Ethical approval was granted by the NRES Committee West Midlands - The Black Country (REC 12/WM/0258).

## Results

Overall 790 patients were included in the study. Baseline age, sex, smoking status, BMI and symptom duration are shown in Table 1. There were significant differences noted between groups for age and symptom duration (*p* < 0.001 for both). The median duration of symptoms at time of clinic visit was 26 days, however there was significant variation (IQR 13.3–54.4). Data on ethnicity were also recorded; 60.7% of patients considered themselves as White British, 20.4% Asian or Asian British and 7.8% Black or Black British.

**Table 1:** Baseline patient characteristics

	All	RA	UIA	CSA	Other
**No. patients**	790	368	139	169	114
Age*, years mean (SD)	52.4 (15.5)	55.5 (15.2)	51.4 (15.4)	46.3 (13.7)	52.3 (16.5)
*Missing (no.)*	*n = 0*	*n = 0*	*n = 0*	*n = 0*	*n = 0*
**Sex**, % female	64.3	68.8	59.0	72.8	43.9
*Missing (no.)*	*n = 0*	*n = 0*	*n = 0*	*n = 0*	*n = 0*
**Ethnicity**,%					
White British	60.7	58.2	59.7	60.4	71.1
White Other	4.7	4.6	3.6	6.6	3.5
Asian or Asian British	20.4	20.4	22.3	21.9	15.8
Black or Black British	7.8	9.0	10.8	4.8	5.3
Other	2.7	2.8	2.8	3.6	0.9
*Missing or not stated (no.)*	*n = 27*	*n = 18*	*n = 1*	*n = 4*	*n = 4*
**Smoking status**, %					
Current	19.9	20.5	18.7	21.3	18.4
Previous	31.5	29.9	34.5	28.4	37.8
Never	47.2	49.2	45.3	49.7	43.2
* Missing (no.)*	*n = 8*	*n = 2*	*n = 2*	*n = 1*	*n = 3*
**BMI**, kg/m^2^ mean (SD)	28.7 (6.4)	28.7 (6.5)	28.0 (5.6)	28.9 (7.1)	29.5 (6.1)
*Missing (no.)*	*n = 41*	*n = 24*	*n = 5*	*n = 8*	*n = 4*
Symptom duration*,days median (IQR)	26.0 (13.3–54.4)	24.1 (13.0–52.2)	24.1 (13.0–57.0)	34.0 (18.0–70.1)	25.7 (10.5–55.7)
*Missing (no.)*	*n = 12*	*n = 7*	*n = 1*	*n = 2*	*n = 2*

*C* current, *CSA* clinically suspect arthralgia, *N* never, *no*. number of cases, *P* previous, *RA* rheumatoid arthritis, *SD* standard deviation, *UIA* unclassified inflammatory arthritis

* denotes statistically significant difference between groups (*p* < 0.001, Kruskall Wallis test)

**Fig. 1:**
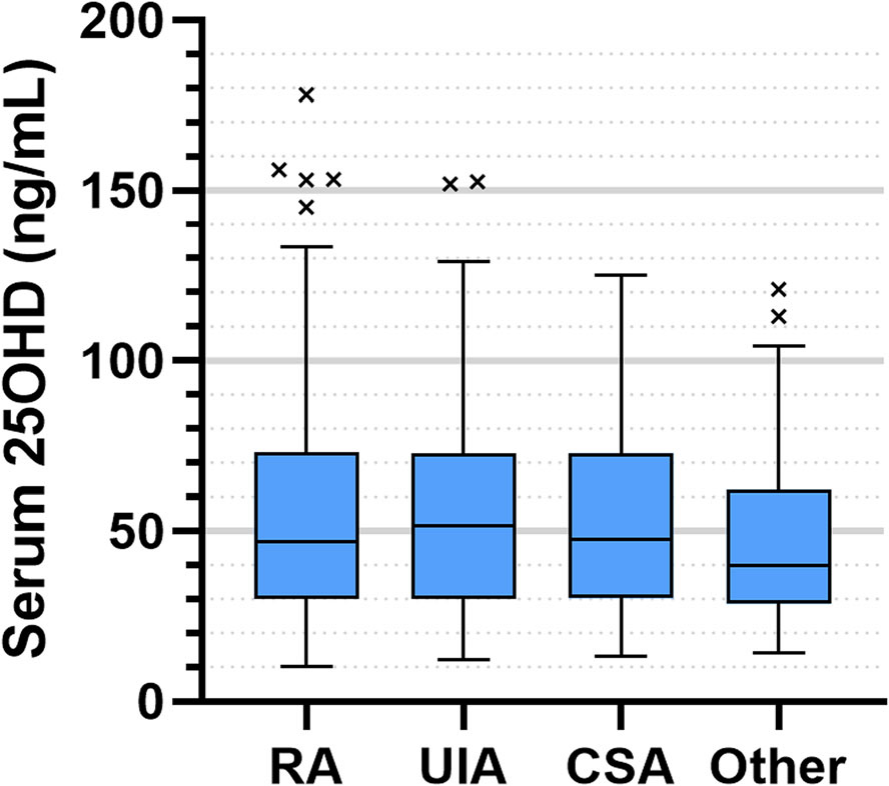
Concentrations of serum 25OHD levels across diagnostic groups where levels were available at baseline. Boxes represent median and interquartile range (IQR), whiskers represent the range, and beyond this any extreme outlier values are also shown. Values for each group are as follows: (i) rheumatoid arthritis (RA), *n* = 335, median 46.7, IQR 30.0–73.0; (ii) undifferentiated inflammatory arthritis (UIA), *n* = 130, median 51.5, IQR 30.0–72.7; (iii) clinically suspect arthralgia (CSA), *n* = 156, median 47.7, IQR 30.3–73.0; (iv) other forms of arthritis (Other) *n* = 102, median 39.9, IQR 28.6–62.2

We initially compared 25OHD levels between clinical groups [RA, UIA, CSA and other diagnoses (e.g. psoriatic arthritis, gout)] at initial clinical presentation to a Rheumatologist and prior to the commencement of disease modifying anti-rheumatic drug (DMARD) therapy for the patient’s arthritis where clinically indicated. There were no significant differences between groups (Fig. 1). However, there was a wide distribution of 25OHD values across all groups [median IQR (ng/mL): RA = 46.7 (30.0–73.0), UIA = 51.5 (30.0–72.7), CSA = 47.7 (30.3–73.0), Other = 39.9 (28.6–62.2)]. Of note, a significantly higher number of RA patient samples were taken from March to September (Suppl Table 1), a time of year when most adults will be able to synthesise vitamin D from sunlight [18]. In the RA group, those tested between March and September had on average a higher baseline vitamin D, although this was not statistically significant (Suppl Fig. 1, *p* = 0.165).

Subsequently, we analysed whether serum 25OHD levels correlated with clinical disease activity measures and other disease related variables in patients diagnosed with RA at baseline. 25OHD levels were recorded for 335 of these patients; there were no clear associations between serum 25OHD levels and baseline EMS duration, TJC, SJC, DAS28-ESR, DAS28-CRP, VAS pain, VAS fatigue, VAS EMS, PHQ9, HAQ, FACIT-Fatigue, CRP or ESR (Table 2).

**Table 2:** Correlations between serum 25OHD concentrations at baseline and clinical characteristics of RA patients at baseline

	No. patients	Correlation co-efficient	*P* value
**Duration EMS**	335	0.074	0.191
**ESR**	335	−0.045	0.418
**CRP**	321	−0.015	0.785
**TJC68**	333	0.075	0.170
**SJC66**	333	0.029	0.601
**TJC28**	333	0.077	0.163
**SJC28**	333	0.017	0.758
**VAS Patient global**	327	−0.008	0.157
**DAS28-ESR**	315	0.037	0.513
**DAS28-CRP**	324	0.047	0.402
**VAS pain**	319	0.012	0.831
**VAS EMS**	115	−0.103	0.272
**VAS fatigue**	319	−0.021	0.713
**FACIT fatigue**	287	0.005	0.926
**HAQ**	286	−0.014	0.808
**PHQ9**	297	−0.006	0.913

*CRP* C-reactive protein, *DAS* disease activity score, *EMS* early morning stiffness, *ESR* erythrocyte sedimentation rate, *FACIT* functional assessment of chronic illness therapy, *HAQ* health assessment questionnaire, *PHQ* patient health questionnaire, *SJC* swollen joint count, *TJC* tender joint count, *VAS* visual analogue scale

**Fig. 2:**
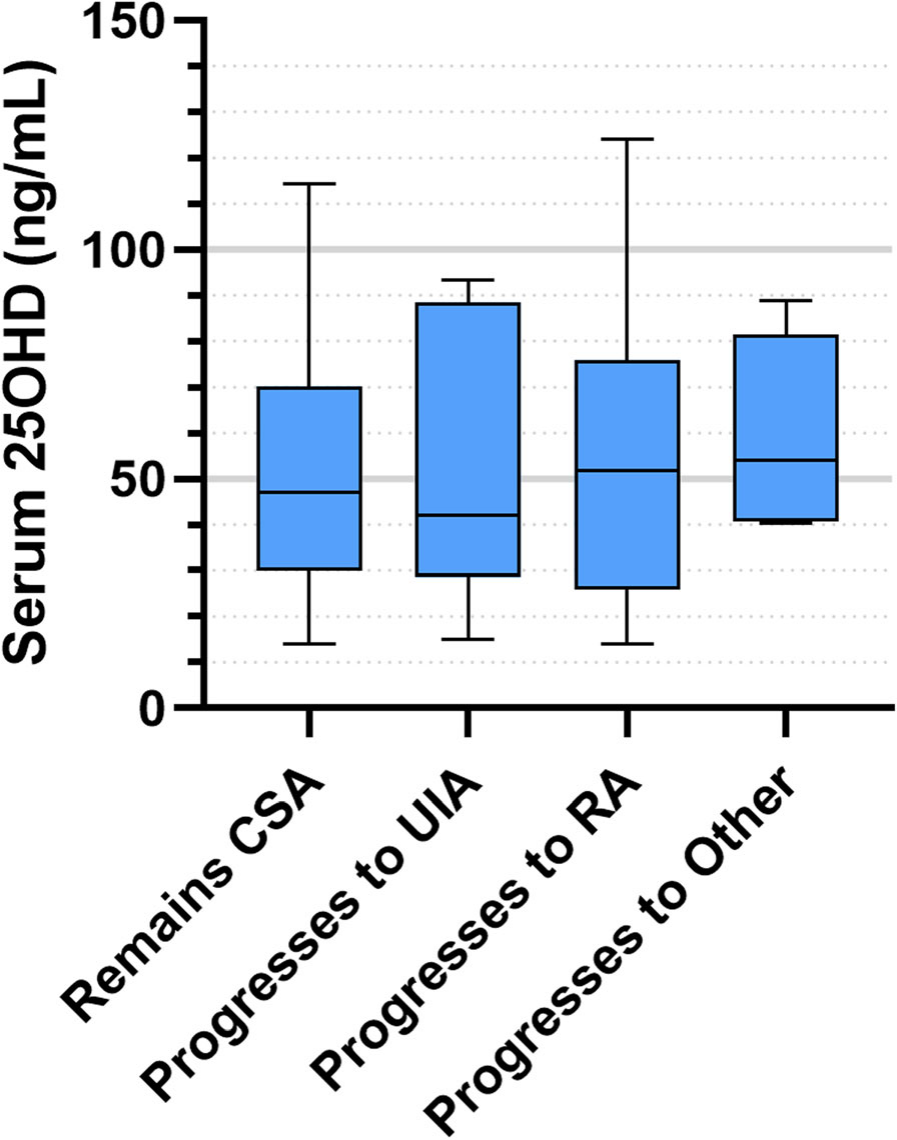
Concentrations of serum 25OHD for all CSA patients at baseline according to final diagnosis after 12 months. Boxes represent median and interquartile range (IQR), whiskers represent the range. Values for each group are as follow: (i) Remained CSA, *n* = 60, median 47.1, IQR 29.6–68.8; (ii) Progressed to UIA, *n* = 11, median 42.0, IQR 28.5–88.5; (iii) Progressed to RA, *n* = 28, median 51.8, IQR 25.9–76.0; (iv) Progressed to Other; *n* = 6, median 54.0, IQR 40.8–81.5. There were no statistically significant differences between groups (*p* = 0.851)

**Fig. 3:**
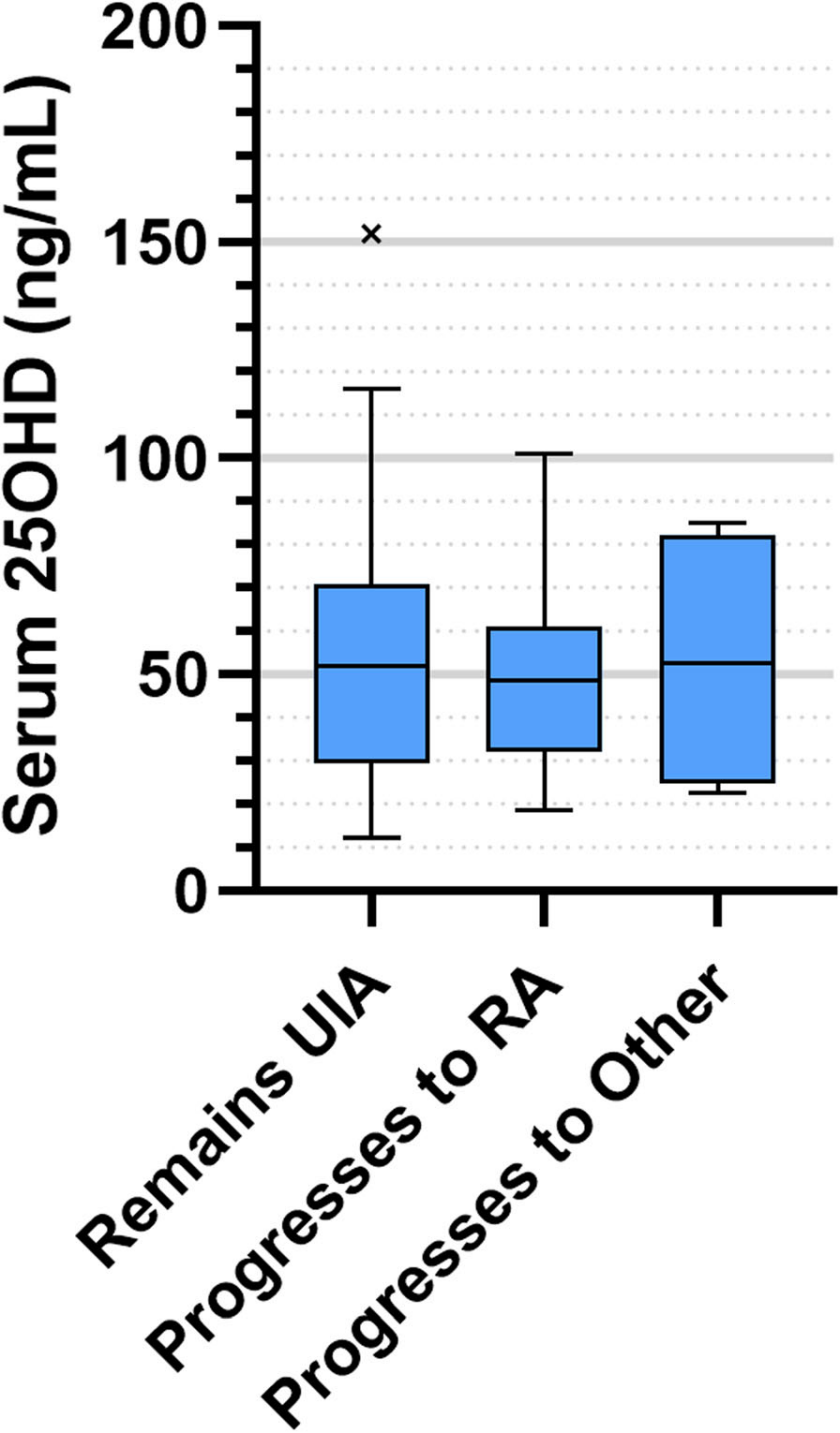
Concentrations of serum 25OHD for all UIA patients at baseline according to final diagnosis after 12 months. Boxes represent median and interquartile range (IQR), whiskers represent the range, and beyond this any extreme outlier values are also shown. Values for each group are as follows: (i) Remained UIA, *n* = 58, median 52.0, IQR 29.5–71.0; (ii) Progressed to RA (*n* = 19, median 48.8, IQR 32.0–61.0); (iii) Progressed to Other; *n* = 4, median 52.5, IQR 24.7–82.3. There were no statistically significant differences between groups (*p* = 0.908)

Finally, we took CSA and UIA patients who had been enrolled in the study for at least 12M, and compared baseline serum 25OHD levels in those who progressed to other diagnoses at 12 M with those whose diagnosis remained unchanged. For the CSA group (*n* = 150), follow-up data were not available for 36 patients (24%), and a further 9 patients did not have vitamin D levels measured at baseline, leaving 105 patients eligible for inclusion in the subgroup analysis. Of these, 60 remained CSA, 28 progressed to RA, 11 progressed to UIA and 6 progressed to other forms of inflammatory arthritis. Baseline vitamin D levels were not significantly different between groups (*p* = 0.851, Fig. 2). In the UIA group (*n* = 120), follow-up data were not available for 35 patients (28.9%), and vitamin D data were missing for a further 4 patients, leaving 81 patients eligible for inclusion in this subgroup analysis. Of these 81 patients, 58 patients remained UIA, 19 progressed to RA and 4 progressed to other forms of chronic inflammatory arthritis. Vitamin D levels were not significantly different between those who remained UIA, and those who progressed to RA or other diagnoses (*p* = 0.908, Fig. 3). Importantly, in the CSA and UIA groups, 25OHD levels at baseline were not significantly different in those without follow up data compared with those for whom follow up data were available (Suppl. Figure 2, *p* = 0.863).

Some CSA and UIA patients were commenced on DMARDs (methotrexate, hydroxychloroquine or sulfasalazine, alone or in combination) during follow-up if the treating clinician felt that was appropriate. Progression of either CSA or UIA to RA may be influenced by DMARD therapy commenced during the clinical follow up period as this may reduce rate of progression to RA in those at risk. Of the CSA patients who remained as having CSA, only one (baseline vitamin D level 68 ng/mL) had been commenced on a DMARD. Of the CSA patients who progressed to RA, eight (baseline vitamin D level 66.5 ng/mL (48.0–79.5)) had been commenced on a DMARD before RA development. In the CSA population, DMARD use during follow-up is thus unlikely to be a factor limiting transition to RA. Of the UIA patients who remained UIA, 27 had been commenced on a DMARD (baseline vitamin D level 46.9 ng/mL (28.3–67.0)) while of those who progressed to RA, 14 had been commenced on a DMARD before RA developed (baseline vitamin D level 50.1 ng/mL (32.0–60.0)). It is possible that some of the UIA patients on DMARDs who remained UIA during follow-up may have actually developed RA had they not commenced a DMARD. However, of those UIA patients started on a DMARD, there was no difference in the baseline vitamin D level between those who progressed to RA and those who did not.

## Discussion

This study exploring the relationship between vitamin D levels and clinical characteristics at presentation and final clinical diagnoses after follow up is, to our knowledge, the first of its kind to be conducted in an early arthritis cohort. Furthermore, we assessed more disease related variables than previous studies, including markers relating to fatigue (VAS fatigue and FACIT-Fatigue scores). Nielen et al. have previously reported that vitamin D deficiency does not increase risk of progression to RA in patients at risk on the basis of positive serology and musculoskeletal symptoms in the absence of synovitis [14]. Similarly, in our cohort, despite there being a range of different 25OHD values in all groups, we did not find any relationship between 25OHD and clinical variables in early RA and the development of arthritis/RA in those at risk with CSA/UIA. This latter finding may be worth further exploration in a larger adequately powered cohort.

The main strengths of this study were the sample size, which is larger than previous early arthritis cohorts in which vitamin D has been analysed, and therefore provides novel insight into vitamin D at this critical time point in the trajectory of inflammatory joint disease. We compared serum 25OHD levels with a wide range of clinical and biochemical markers of disease, including both subjective and objective measures, giving a comprehensive insight into their relationship with serum 25OHD. Previous studies have found conflicting results as to whether there is a relationship between vitamin D levels and markers of disease activity [19]. Data presented in the current study strongly suggest that low serum 25OHD is not linked to any marker of disease activity in early RA. Furthermore, the inclusion of patients with UIA provides a novel insight into this facet of arthritis.

There are important limitations to our data. Firstly, although the study had large numbers of patients, there was significant loss to follow up in all groups at 12 months. This reduced power to detect significant differences in 25OHD levels between those UIA and CSA patients who progressed to RA and those who did not progress to RA. Secondly, a significantly higher number of RA patient samples were taken from March to September; a time of year when most adults will be able to synthesise vitamin D from the sunlight [18]. In the RA group those tested between March and September had on average a higher baseline vitamin D, although this was not statistically significant. Nevertheless, this could have impacted on our ability to detect any relationship between low 25OHD and markers of RA disease activity. In addition, we were unable to reliably determine which patients were receiving vitamin D supplements, and when they may have begun these in relation to the onset of their joint symptoms, owing to the frequent (and inconsistently recorded) use of over-the-counter (OTC) supplements. GPs are encouraged to recommend OTC supplements to patients with vitamin D insufficiency (30-50 nmol/L serum 25OHD) rather than issuing a prescription for vitamin D [18]. Accordingly, GP records could not be used as a means of determining which patients were taking vitamin D supplements at baseline. Many patients do not tell doctors they take vitamin supplements as they do not necessarily consider them medications, and furthermore, patients are usually unaware of their preparation’s formulation (vitamin D2 vs D3) or strength making it impossible to adjust for vitamin D supplementation use in our analysis.

This study focused on total serum 25OHD concentrations as this is the analysis used in most routine assay services. However, there are multiple vitamin D metabolites which affect both innate and adaptive immune processes at the molecular level, and these have been shown to play a role in the aetiopathogenesis of RA. Studies have shown the active form of vitamin D, 1,25(OH)_2_D, promotes tolerogenic DC cell function [20, 21], maintains the balance of Th1/Th2 cells [22], suppresses the proinflammatory TH17 phenotype [23–25], and promotes T-regulatory (Treg) cell function [26, 27], whilst facilitating migration of T cells to the site of inflammation and phenotype stabilisation [28–30]. Similarly, the vitamin D receptor (VDR), which plays an important role in facilitating PBMC responses to 1,25(OH)_2_D in immune homeostasis [31], is altered in RA [32] and VDR polymorphisms are linked to the development of arthritis [33]. Thus, it is likely that simple measurement of serum 25OHD provides only a limited perspective on the potential impact of vitamin D on diseases such as RA. Further studies are needed to determine if other forms of vitamin D are more effective determinants of its anti-inflammatory action.

The compartment in which vitamin D is measured may also be important. Serum can be obtained through simple phlebotomy, but the levels and behaviour of vitamin D and its metabolites at the site of inflammation, the synovial joint, may differ relative to peripheral blood. Our group has reported differences in vitamin D and its metabolites between serum and synovial fluid in patients with established RA [34]. Although we did not measure cytokine expression, evidence from in vitro and murine studies suggests the differences inflammatory cytokine milieu in the synovial tissue compartment will modulate the effects of vitamin D further [35]. Thus, future studies should focus on analysis of multiple vitamin D metabolites and their relationship with inflammatory factors within specific microenvironment sites of RA disease. Further research is thus needed to better elucidate the role of vitamin D and its metabolites not only in the peripheral blood, but at the site of inflammation, the synovial joint. Understanding how vitamin D changes with, and as a result of, the inflammatory milieu in the synovial joint will provide new insights, and potential novel treatment approaches. In recent studies of T cells from RA patients we observed much stronger responses to 1,25(OH)_2_D in T cells from the peripheral blood compared to paired inflamed joint synovial fluid from the same patient [36]. This suggests that once RA disease is established, there is insensitivity to 1,25(OH)_2_D in immune cells, with this effect severely limiting the anti-inflammatory benefits of 1,25(OH)_2_D. Thus, it is possible that, even in early RA disease, much higher levels of vitamin D than previously thought are required to induce beneficial effects on synovial inflamamtion.

## Conclusion

This study did not show any clear associations between serum 25OHD and baseline diagnosis, in patients with a range of different inflammatory arthritides, disease activity in patients with RA, or progression from UIA or CSA to RA. Our data thus provide further clarity on an issue where previous studies have yielded conflicting data; that is, whether RA disease activity is linked to serum vitamin D levels. Future research should focus on validating these findings including those related to vitamin D at baseline and subsequent disease trajectories, in another cohort powered to detect statistical significance. Furthermore, it will be important to assess different vitamin D metabolites, their carrier proteins, and enzymes involved in vitamin D metabolism that might affect the local action of vitamin D in the joint, to allow a more comprehensive understanding of the role of vitamin D in RA pathogenesis.

## Supplementary information

**Additional file 1: Suppl. Table 1.** Number of patients in each diagnostic group who had their serum 25OHD measured between March and September. **Suppl. Figure 1.** Baseline serum 25OHD levels in RA patients taken at different times of the year. Boxes represent median and interquartile range (IQR), whiskers represent the range, and beyond this any extreme outlier values are also shown. Median serum 25OHD for the Mar-Sept group was 50.0, IQR 31.5–73.6. For the Oct-Feb group, median serum 25OHD3 was 42.0, IQR 25.8–71.5. The difference between groups was not statistically significant (*p* = 0.126). **Suppl. Figure 2.** Boxplot comparing 25OHD between those who did and did not attend follow up at or beyond 12 M [*n* = 530 (75.3%) and *n* = 174 (24.7%) respectively]. Boxes represent median and interquartile range (IQR), whiskers represent the range, and beyond this any extreme outlier values are also shown. Median serum 25OHD for those Lost to follow up (LTFU) was 47.9, IQR 30.0–72.8. Median serum 25OHD for those not LTFU 48.9, IQR 30.0–73.0. The differences between groups was not statistically significant (*p* = 0.863).

## Data Availability

All data generated or analyzed during this study are included in this published article [and its supplementary information files] or are available from the corresponding author on reasonable request.
