# Supplementary material for: Vitamin D and early rheumatoid arthritis
Source: BMC Rheumatol. 2020 Jul 27;4:38. doi: 10.1186/s41927-020-00134-7 (PMC7384217; doi:10.1186/s41927-020-00134-7)
Supplement: Supplementary file 1 — Additional file 1: Suppl. Table 1. Number of patients in each diagnostic group who had their serum 25OHD measured between March and September. Suppl. Figure 1. Baseline serum 25OHD levels in RA patients taken at different times of the year. Boxes represent median and interquartile range (IQR), whiskers represent the range, and beyond this any extreme outlier values are also shown. Median serum 25OHD for the Mar-Sept group was 50.0, IQR 31.5–73.6. For the Oct-Feb group, median serum 25OHD3 was 42.0, IQR 25.8–71.5. The difference between groups was not statistically significant (p = 0.126). Suppl. Figure 2. Boxplot comparing 25OHD between those who did and did not attend follow up at or beyond 12 M [n = 530 (75.3%) and n = 174 (24.7%) respectively]. Boxes represent median and interquartile range (IQR), whiskers represent the range, and beyond this any extreme outlier values are also shown. Median serum 25OHD for those Lost to follow up (LTFU) was 47.9, IQR 30.0–72.8. Median serum 25OHD for those not LTFU 48.9, IQR 30.0–73.0. The differences between groups was not statistically significant (p = 0.863). [file 41927_2020_134_MOESM1_ESM.docx]

**Supplementary figures and tables**

**Suppl. Table 1.** Number of patients in each diagnostic group who had their serum 25OHD measured between March and September

| **Diagnostic Group** | **Number of patients whose vitamin D levels were measured Mar-Sept**  *number of patients (%)* | **Number of patients whose vitamin D levels were measured Oct-Feb**  *number of patients (%)* |
| --- | --- | --- |
| RA | 215 (66.6) | 108 (33.4) |
| UIA | 71 (58.2) | 51 (41.8) |
| CSA | 79 (52.7) | 71 (47.3) |
| Other | 60 (52.6) | 54 (47.4) |

*Percentages are rounded to one decimal place. Difference in numbers between each group was statistically significant (p=0.005, X_2_ test). CSA = Clinically Significant Arthralgia; RA = Rheumatoid Arthritis; UIA = Undifferentiated Inflammatory Arthritis.*


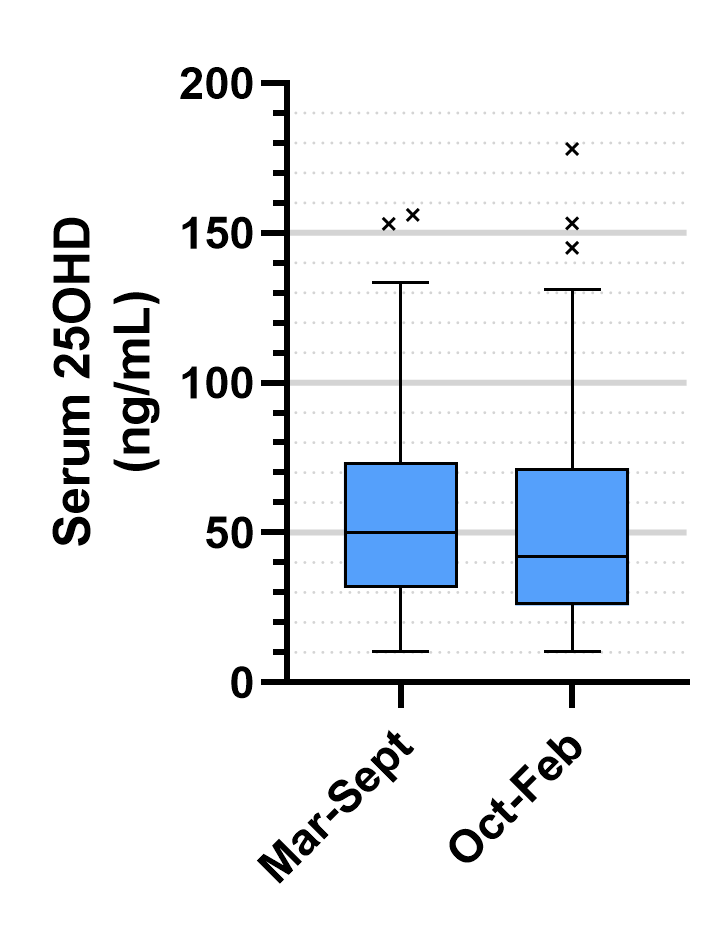


**Suppl. Figure 1. Baseline serum 25OHD levels in RA patients taken at different times of the year.**  Boxes represent median and interquartile range (IQR), whiskers represent the range, and beyond this any extreme outlier values are also shown. Median serum 25OHD for the Mar-Sept group was 52.7, IQR 33.0-76.0. For the Oct-Feb group, median serum 25OHD3 was 43.3, IQR 28.0-69.0. The difference between groups was not statistically significant (p=0.165).


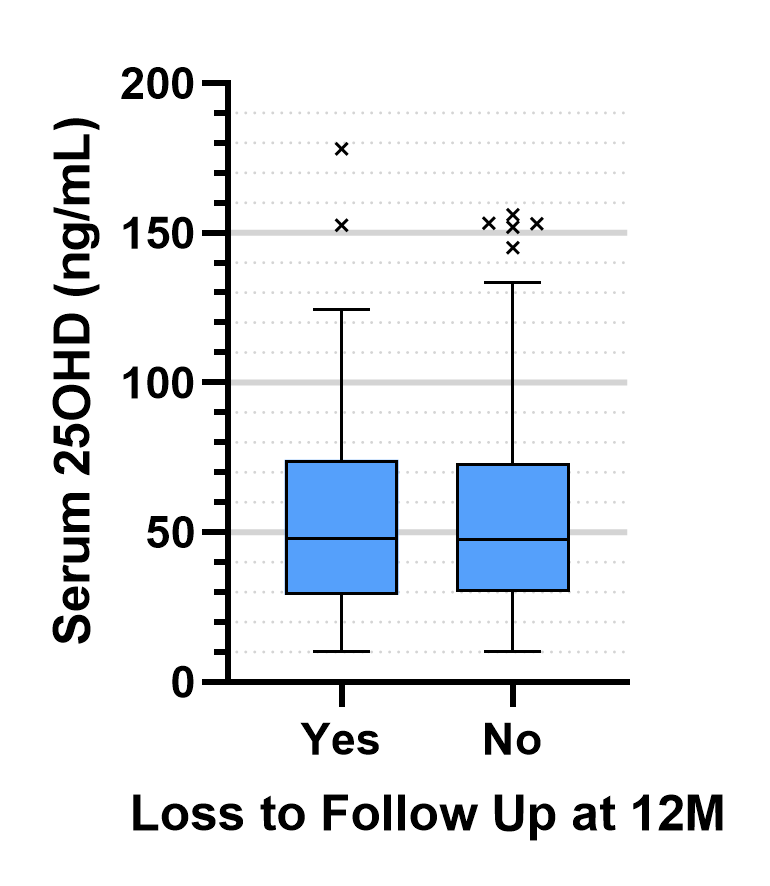


**Suppl. Figure 2.** Boxplot comparing 25OHD between those who did and did not attend follow up at or beyond 12M [n=530 (75.3%) and n=174 (24.7%) respectively]. Boxes represent median and interquartile range (IQR), whiskers represent the range, and beyond this any extreme outlier values are also shown. Median serum 25OHD for those Lost to follow up (LTFU) was 47.9, IQR 30.0-72.8. Median serum 25OHD for those not LTFU 48.9, IQR 30.0-73.0. The differences between groups was not statistically significant (p=0.863).
